# Incidence of lung cancer and air pollution in boroughs of Chile: an ecological study

**DOI:** 10.3332/ecancer.2021.1247

**Published:** 2021-06-10

**Authors:** Jorge Sapunar-Zenteno, Pedro Ferrer-Rosende, Christian Caglevic

**Affiliations:** 1 Research and Teaching Unit, Department of Cancer Research, Fundación Arturo López Pérez Cancer Institute, Cano y Aponte 927, Providencia, Santiago 7500000, Chile; 2 Internal Medicine Department, School of Medicine, Universidad de La Frontera, Manuel Montt 112, Temuco, La Araucanía 4780000, Chile; 3 Department of Cancer Research, Fundación Arturo López Pérez Cancer Institute, Cano y Aponte 927, Providencia, Santiago 7500000, Chile

**Keywords:** lung cancer, epidemiology, environmental air pollutants

## Abstract

Lung cancer frequency has been progressively increasing; this has been linked to the use of inhaled tobacco and air pollution. In Chile, air pollution has reached alarming levels due to motor vehicle traffic, firewood burning for heating and minerals in urban areas; for this reason, our objective was to evaluate the association between the incidence of lung cancer and the concentration of the main air pollutants monitored in the country. We carried out a cross-sectional ecological study that evaluated the association between the average incidence of lung cancer in a 5-year period (2015–2019) with the average annual concentration of six atmospheric pollutants in the 5 years prior in 14 Chilean boroughs, using the population of beneficiaries of the Fundación Arturo-López-Pérez Cancer Institute. The annualised incidence of lung cancer was 9.77 per 100,000 and it varied significantly within the boroughs studied. When evaluating the relationship between lung-cancer incidence and the average concentration of atmospheric pollutants, we only found a direct and significant correlation between the level of respirable particulates 2.5 and the incidence of adenocarcinomas (*β*: 0.16; *p*: 0.023).

## Introduction

Since 1950, there has been an increasing trend in the incidence of lung cancer worldwide, especially in high-income countries according to the World Bank classification. This increase has been related to different factors, but causality has been established in two of them: the consumption of inhaled tobacco and environmental pollution, mainly by inhalable particulate material. The association between lung cancer and air pollution is in addition to the well-known relationship between this factor and non-neoplastic cardiovascular and respiratory diseases [1].

The evidence linking air pollution with the development of lung cancer has been obtained mainly in the last 40 years due to systematic monitoring of air quality and the development of ecological studies [2]. Pope’s reports have shown since the beginning of this millennium that for every 10 μg/m^3^ of air contaminated with fine particles, the risk of developing lung cancer increases by approximately 8% [3]. A more recent British study shows that living within 50 m of a London motorway increases the risk of developing lung cancer by almost 10% [4].

Chile is no exception to the global problem of environmental pollution in general and air pollution in particular. In the city of Santiago, the country’s capital with a population of around 7.5 million inhabitants, the problem of air pollution has been critical since the 1980s [5]. Due to the growth of other urban areas with poor natural ventilation conditions, the increase in vehicular traffic, industrial activity and the use of polluting fuels for heating, the problem of air pollution has progressively spread to other Chilean regions.

In 2020, approximately 1,800,000 people are expected to die from lung cancer worldwide [6], it being by far the leading cause of cancer death in high-, low- and middle-income countries. Epidemiological data of lung cancer in Chile reflect a late diagnosis, which adds to the high mortality of this disease, unless it is diagnosed in very early stages. According to the projections of the International Agency for Research on Cancer, published on the Global Cancer Observatory (GLOBOCAN) portal [7], 3,873 new cases and 3,581 deaths from lung cancer were estimated for Chile in 2018. The Antofagasta Region, in northern Chile, is one of the areas with the highest incidence of lung and airway cancers in the world. In this particular case, the incidence rate has been linked to arsenic contaminant in the water, although it is possible that other environmental pollutants may explain the incidence and mortality from lung cancer in this geographical area [8].

Death rates from lung cancer are higher in men than in women. In Chile, age-adjusted mortality rate in the period 1990–2015 was higher in the northernmost regions (Antofagasta, Tarapacá, Arica-Parinacota and Atacama in decreasing order), while it was lower in the Araucanía, Biobío and Maule regions in the south, both for men and women [9].

This study aims to evaluate whether there is a demonstrable association between the incidence of lung cancer and the concentration of the main air pollutants monitored in Chile through an ecological study based on epidemiological data from patients from the Fundación Arturo López Pérez Cancer Institute in Santiago, Chile residing in one of 14 boroughs selected for their good records of the presence of six atmospheric pollutants. Chile has a network of air-quality monitoring stations located in the main cities or in places where productive activities such as mining, heavy industry and thermoelectric power generation generate pollution. This information is centralised through the Ministry of the Environment and reported through the National Air-Quality Information Service, which gives us reliable sources for the analysis of our data and their correct interpretation.

## Subjects and methods

An ecological cross-sectional study to evaluate the association between annualised incidence of lung cancer in the period 2015–2019 and the average annual concentration of six air pollutants in 14 Chilean boroughs during the 5 years prior to the estimate.

The study population was selected among the captive subjects of a Chilean health centre dedicated to cancer management offering cancer-related insurance to potential patients, covering diagnostic and treatment costs of neoplastic events. The Arturo López Pérez Foundation (FALP) Cancer Institute meets these characteristics.

After approval by the FALP Scientific Ethics Committee, we obtained the distribution by age, sex and borough of the beneficiaries of the cancer insurance, as well as the new cases of lung cancer treated at the Institute in 2015, 2016, 2017, 2018 and 2019. Only histologically confirmed lung cancer cases among insurance beneficiaries were considered to estimate its incidence.

In order to correct the potential biases implicit in projecting the incidence of lung cancer to the entire Chilean population based on the population of FALP-insurance beneficiaries, this rate was adjusted for the age distribution of the 2012 census using the direct method. To control the effect of age distribution among beneficiaries from each borough on the association between lung cancer incidence and pollutant concentration, we adjusted the borough rate for the proportion of beneficiaries in the highest-risk age group.

Total and by-sex incidence rates were estimated with their 95% confidence interval based on gamma distribution. The relative importance of the main histological types of lung cancer is described.

The average annual concentration of air pollutants was obtained from the data recorded by air-quality monitoring stations throughout Chile. The pollutants studied were respirable particulates 2.5 and 10 (PM_2.5_, PM_10_), sulphur dioxide (SO_2_), nitrogen monoxide (NO), nitrogen dioxide (NO_2_) and ozone (O_3_). Techniques for determining the concentration of atmospheric pollutants are available on the website of the National Air Quality Information System [16]. We considered the average of the last 5 years prior to the incidence estimate due to the lack of validated data in oldest records.

To evaluate the association between lung cancer incidence and the average concentration of air pollutants, we selected boroughs equipped with active monitoring stations and validated registries that have more than 5,000 FALP-insurance beneficiaries. Considering the relative importance of adenocarcinoma among lung cancers, we also analysed the correlation between this histological type and average pollution by PM_2.5_ and PM_10_; SO_2_; NO; NO_2_ and O_3_.

Statistical analysis was performed with the R program version 3.6.0 (R Core Team, 2018. Vienna, Austria). Using multiple linear regression, we evaluated the effect of air pollution on the incidence rate of lung cancer and lung adenocarcinoma, all as continuous variables, considering age and Human Develop Index (HDI) by commune. We previously checked the linearity of the relationship between the lung cancer incidence rate and the average concentration of pollutants. Both variables were normally distributed in the communes. Covariates for the model were HDI, age, and mean pollutant concentrations.

## Results

**Figure 1.**
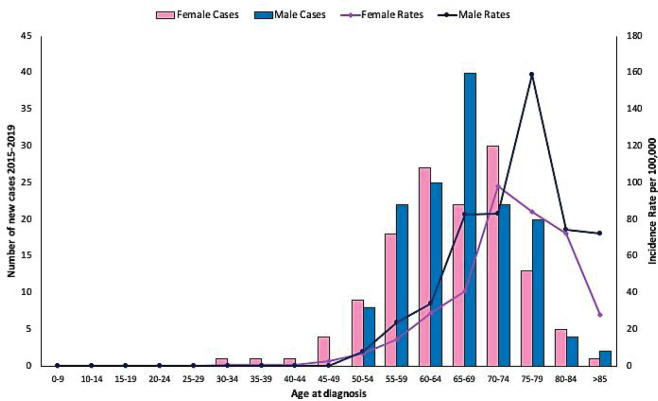
Age distribution by sex expressed in 5-year periods for lung cancer cases occurring in beneficiaries of FALP cancer insurance in the period 2015–2019.

**Figure 2.**
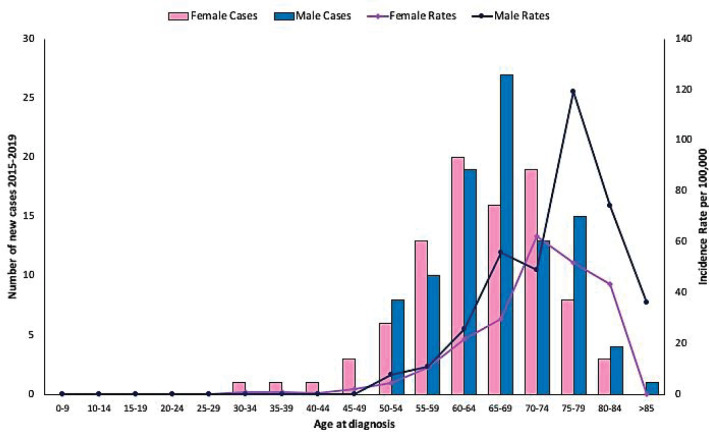
Age distribution by sex expressed in 5-year periods for lung cancer adenocarcinoma cases occurring in beneficiaries of FALP cancer insurance in the period 2015–2019.

During the period 2015–2019, 275 new histologically-confirmed lung-cancer cases were treated in people with FALP cancer insurance. The annualised incidence of lung cancer was 9.77 per 100,000 people FALP insurance beneficiaries (95% CI: 8.7–11.0). Table 1 shows incidence by sex, adjusted according to projections from the 2012 census, highlighting a significantly higher rate in men (11.1 versus 8.68). Figure 1 shows age distribution by 5-year periods of lung cancer cases, which is more frequent between 55 and 79 years of age. The highest incidence in men was observed between 75 and 79 years, while in women it was between 70 and 74 years; in both cases, it was more than 10 times higher than the general rate. Table 2 shows distribution by histological type according to sex and mean age, adenocarcinoma being the most frequent type (68.4%), followed by squamous carcinoma (17.1%). Figure 2 shows age distribution by 5-year periods of the cases of lung adenocarcinoma, which did not differ from what was observed in lung cancer in general.

Table 3 shows the annualised incidence of lung cancer in 14 Chilean boroughs with more than 5,000 FALP cancer-insurance beneficiaries; data are presented both raw and adjusted for the proportion of people in the highest-risk age group. The boroughs with the highest adjusted incidence were Talca in the Maule Region (south), Las Condes in Greater Santiago (centre) and Los Angeles in the Bío-Bío Region (south). Table 4 shows the annualised incidence of lung adenocarcinoma in the same boroughs, with the highest adjusted rates registered for La Condes, Los Ángeles and Puente Alto (centre). It is noteworthy that in some boroughs, the incidence adjusted by age group at risk was extremely different between the sexes, with males predominating in some boroughs, such as Antofagasta (21.25 versus 2.01 cases per 100,000 in males and females, respectively), and the opposite occurring in others, such as Santiago (18.6 versus 5.55 cases per 100,000 in females and males, respectively).

The association between lung cancer incidence rate and the average concentration of six atmospheric pollutants, estimated by multiple lineal regression, is presented in Table 5. We only found an inverse association between the incidence of lung cancer and the concentration of SO_2_ in men, which loses its significance when adjusting for age and HDI. Table 6 shows the association between lung adenocarcinoma incidence rate and the average concentration of six atmospheric pollutants, estimated by multiple lineal regression. Again, we found an inverse association between the incidence of adenocarcinoma and the SO_2_ concentration, which in this case loses significance when we adjust for HDI. We found a direct association between the incidence of adenocarcinoma in the general population and the concentration of O_3_, which is lost when adjusting for age and HDI. Finally, we found a direct and significant association between the incidence of lung adenocarcinoma and the PM_2.5_ concentration, when adjusting for HDI.

## Discussion

According to GLOBOCAN estimates, lung cancer accounted for 11.6% of new cancer cases worldwide in 2018. The mean yearly incidence of this disease in countries with a high HDI was 40.4 cases per 100,000 males and 19.1 cases per 100,000 females. In countries with medium and low HDI, yearly rates were 11.8 cases per 100,000 males and 4.6 cases per 100,000 females. For South America, GLOBOCAN estimated 16.8 cases per 100,000 men per year and 10.2 cases per 100,000 women per year [11]. In Chile, crude airway- and lung-cancer incidence rates for the 5-year period 2003–2007, estimated from the registry of sentinel centres, were 17.1 cases per 100,000 men and 9.9 cases per 100,000 women [9]. Our study is the first one to directly obtain a lung cancer incidence rate for a Chilean population: it was 11.1 cases per 100,000 males per year and 8.68 cases per 100,000 females per year, a figure in the range observed for countries with medium to lower HDI in GLOBOCAN projections. The possible demographic differences between the population with FALP cancer insurance and the general Chilean population were partially corrected by adjusting the rate for the age distribution of the general population as registered in the 2012 census. On the other hand, FALP cancer insurance is mainly subscribed by companies for their workers, which would reduce the risk of selection bias given by individual affiliation.

Regarding the distribution by histological type, this is similar to the results of large series from the United States and Latin America [12, 13], with adenocarcinoma followed by squamous carcinoma.

In the 14 boroughs with a population of over 5,000 FALP insured selected for analysis, we observed a great variation in the mean annualised incidence of lung cancer ranging from 4.6 cases per 100,000 people per year in Viña del Mar, Valparaíso Region (centre) to 15.51 cases per 100,000 people per year in Talca, Maule Region (south). This finding is repeated in the analysis by sex. This variability in incidence by borough suggests that using sentinel centres to estimate incidence is debatable. Among the determinants of this variability, there would be socio-economic factors as shown by GLOBOCAN projections [11].

When assessing the association between the incidence of lung cancer in general and lung adenocarcinoma in particular and the average annual concentration of six air pollutants in the previous 5 years, we found an inverse association with the concentration of SO_2_, that is lost when fitting the linear regression model for age and HDI. We found a direct association between the incidence of adenocarcinoma and the O_3_ concentration that was also lost when the model was adjusted. This pollutant is not emitted directly into the atmosphere but is produced by chemical reactions between volatile organic compounds and nitrogen oxides in the presence of sunlight. Its production is stimulated by luminosity and heat. It is a marker of emissions from vehicles and solvent-related industrial activities [14]. Because O_3_ is a powerful photochemical oxidant, it causes intense irritation to airway conjunctivas and mucosas, which could be classified as an acute effect. No association between the effect of long-term O_3_ exposure and outcomes such as mortality from various causes has been found upon analysis [15]. The borough of Las Condes exhibits the highest average annual O_3_ concentration and has one of the highest adjusted annual mean incidences of lung cancer (14.97 cases per 100,000). It is possible that O_3_ concentration is a marker of exposure to other pollutants with a carcinogenic effect as occurs in breast cancer [16]. The only contaminant that showed a significant direct association with the incidence of adenocarcinoma, when adjusting the model for HDI, was PM_2.5_. A systematic review reported that exposure to high concentrations of both PM_2.5_ and PM_10_ is associated with a slightly increased risk of lung cancer (OR: 1.09; 95% CI: 1.04–1.14 and OR: 1.08; 95% CI: 1.00–1.17, respectively). This association is modified by smoking habits [17]. However, a study carried out in a cohort of non-smokers showed that for every 10 μg/m^3^ increase in the concentration of PM_ 2.5_ the risk of adenocarcinoma increased by 31% [18].

Our study has some limitations due to the lack of validated records of all pollutants from air quality monitoring stations. There is also the possibility of selection bias in the beneficiaries of FALP cancer insurance which may increase the incidence of cancer in relation to the general population. However, the lung cancer incidence rate found is similar to that reported in literature. Although we only selected boroughs with more than 5,000 beneficiaries of FALP cancer insurance, lung cancer projections for some of them were based on few cases, which distorts the subgroup analysis (e.g. by sex). Although literature reports an association between air pollution and risk of lung cancer, the effect is not very high and its confirmation required the accumulation of a large number of individuals in systematic reviews. It is also important to consider the effect of smoking as a recognised risk factor for lung cancer [19] which could act synergistically in the association between other air pollutants and risk of lung cancer [15]. Unfortunately, the magnitude of tobacco consumption by borough in Chile is unknown, so we could not control this variable in the population studied.

## Conclusion

In conclusion, the incidence of lung cancer in the population studied is similar to that of countries with medium and low HDI, in accordance with GLOBOCAN 2018 projections; it is more frequent in males and between 55 and 79 years of age. We found a direct relationship between the frequency of lung adenocarcinoma and the concentration of PM_2.5_ in the air, when adjusting for HDI, considering that this indicator is strongly associated with the incidence of cancer. New studies are required to evaluate the role of smoking in the association between the pollutants studied and lung cancer. Finally, the lessons learned from our study could be applicable in low- and middle-income countries with a similar population.

## Conflicts of interest and funding statement

The authors have no conflicts of interest or funding to declare.

## Figures and Tables

**Table 1.** 2015–2019 lung cancer cases in FALP cancer-insurance beneficiaries and incidence rates adjusted for age and sex according to 2012 census projections.

	Women	Men	Total
Cases 2015–2019	132	143	275
Crude rate 2015–2019 (per 100,000)	8.68 (7.3–10.3)	11.1 (9.3–13.0)	9.77 (8.7–11.0)
Adjusted rate 2015–2019 (per 100,000)	11.42 (9.2–14.0)	12.6 (10.5–15.1)	12.12 (10.5–13.9)

**Table 2.** Histological types of lung cancer occurring in beneficiaries of FALP cancer insurance in the period 2015-2019, by sex and mean age.

Histotype	Frequency (%)			Mean age (±SD)		
	Women	Men	Total	Women	Men	Total
Adenocarcinoma	91 (68.9)	97 (68.3)	188 (68.6)	64.2 ± 9.6	66.9 ± 8.1	65.6 ± 8.9
Squamous-cell Ca	19 (14.4)	28 (19.7)	47 (17.2)	69.9 ± 9.8	69.0 ± 6.6	69.4 ± 8.0
Small-cell lung cancer	13 (9.8)	6 (4.2)	19 (6.9)	65.2 ± 8.1	61.8 ± 6.3	64.1 ± 7.6
NET	6 (4.5)	2 (1.4)	8 (2.9)	66.0 ± 5.7	64.0 ± 7.1	65.5 ± 5.6
Other NSCLC	2 (1.5)	4 (2.8)	6 (2.2)	53.0 ± 1.4	58.5 ± 2.5	56.7 ± 3.5
Sarcomatoid Ca	1 (0.8)	2 (1.4)	3 (1.1)	79	72.5 ± 4.9	74.7 ± 5.1
Large-cell lung cancer	0 (0)	3 (2.2)	3 (1.1)	-	66.0 ± 8.9	66.0 ± 8.9
Total	132	142	274	65.1 ± 9.6	66.8 ± 7.8	66 ± 8.7

Ca, Carcinoma; NET, Tumour neuroendocrino; NSCLC, Non-small-cell lung cancer

**Table 3.** Lung cancer annualised incidence by borough of residence in beneficiaries of FALP cancer insurance between 2015 and 2019, direct and adjusted by risk group.

Borough	Region	Crude rate 2015–2019 (per 100,000)			Adjusted rate 2015–2019 (per 100,000)		
		Female	Male	Total	Female	Male	Total
Antofagasta	Antofagasta	1.62 (0.0–9.0)	17.6 (8.4–32.4)	9.29 (4.6–16.6)	2.01 (0.1–11.2)	21.25 (10.2–39.1)	10.85 (5.4–19.5)
Chillán	Ñuble	6.14 (0.7–22.2)	7.47 (0.9–27.0)	6.74 (1.8–17.3)	5.97 (0.7–21.6)	7.27 (0.9–26.3)	6.57 (1.8–16.8)
Concepción	Biobio	9.74 (2.0–28.5)	11.58 (2.4–33.8)	10.58 (3.9–23.0)	7.86 (1.6–23.0)	9.82 (2.0–28.7)	8.76 (3.2–19.1)
La Florida	Greater Stgo	7.3 (1.5–21.3)	23.23 (10.0–45.8)	14.56 (7.3–26.1)	5.5 (1.1–16.1)	18 (7.7–35.8)	11.24 (5.6–20.2)
La Serena	Coquimbo	14.14 (3.9–36.2)	0.00	7.65 (2.1–19.6)	14.78 (4–37.9)	0.00	7.99 (2.2–20.5)
Las Condes	Greater Stgo	15.39 (6.2–31.7)	24.85 (11.4–47.2)	19.58 (11.2–31.8)	11.81 (4.4–25.5)	18.68 (8.2–36.4)	14.97 (8.3–24.9)
Los Angeles	Biobio	6.68 (0.8–24.1)	19.65 (6.4–45.9)	12.64 (5.1–26.1)	7.66 (0.9–27.7)	21.45 (7.0–50.1)	14 (5.6–28.9)
Puente Alto	Greater Stgo	7.76 (1.6–22.7)	11.65 (3.2–29.8)	9.59 (3.9–19.8)	9.29 (1.9–27.2)	12.79 (3.5–32.7)	10.9 (4.4–22.5)
Puerto Montt	Los Lagos	5.2 (0.6–18.8)	2.99 (0.1–16.7)	4.17 (0.9–12.2)	5.84 (0.7–21.4)	3.8 (0.1–21.2)	4.9 (1.0–14.5)
Punta Arenas	Magallanes	3.78 (0.1–21.0)	8.55 (1.0–30.9)	6.02 (1.2–17.6)	4.19 (0.1–23.4)	8.64 (1.1–31.2)	6.24 (1.3–18.3)
Santiago	Greater Stgo	20.6 (9.4–39.1)	5.86 (0.7–21.2)	14.13 (7.1–25.3)	18.6 (8.5–35.4)	5.55 (0.7–20.1)	12.6 (6.3–22.6)
Talca	Maule	11.67 (3.2–29.9)	20.69 (7.6–45.0)	15.81 (7.6–29.1)	11.46 (3.1–29.4)	20.28 (7.4–44.1)	15.51 (7.4–28.5)
Temuco	La Araucanía	3.08 (0.1–17.2)	7.25 (0.9–26.2)	4.99 (1.0–14.6)	3.07 (0.1–17.1)	7.1 (0.9–25.7)	4.92 (1.0–14.4)
Viña del Mar	Valparaiso	10.76 (2.2–31.5)	0.00	5.87 (1.2–17.2)	8.52 (1.8–24.9)	0.00	4.6 (1.0–13.5)

Stgo, Santiago

**Table 4.** 2015–2019 annualised incidence of lung adenocarcinoma by borough of residence in beneficiaries of FALP cancer insurance; direct and adjusted by risk group.

Borough	Region	Crude rate 2015–2019 (per 100,000)			Adjusted rate 2015–2019 (per 100,000)		
		Female	Male	Total	Female	Male	Total
Antofagasta	Antofagasta	1.62 (0.0–9.0)	7.04 (1.9–18.0)	4.22 (1.4–9.9)	2.01 (0.1–11.2)	8.2 (2.2–21.1)	4.86 (1.6–11.4)
Chillán	Ñuble	3.07 (0.1–17.1)	7.47 (0.9–27.0)	5.06 (1.0–14.8)	2.83 (0.1–15.8)	7.27 (0.9–26.3)	4.87 (1.0–14.3)
Conepción	Biobio	6.49 (0.8–23.5)	7.72 (0.9–27.9)	7.05 (1.9–18.1)	5.24 (0.6–18.9)	6.54 (0.8–23.6)	5.84 (1.6–15)
La Florida	Greater Stgo	7.3 (1.5–21.3)	14.52 (4.7–33.9)	10.59 (4.6–20.9)	5.5 (1.1–16.1)	11.65 (3.7–27.6)	8.33 (3.6–16.5)
La Serena	Coquimbo	7.07 (0.9–25.5)	0.00	3.82 (0.5–13.8)	7.25 (0.9–26.2)	0.00	3.92 (0.5–14.2)
Las Condes	Greater Stgo	13.19 (4.8–28.7)	19.33 (7.8–39.8)	15.91 (8.5–27.2)	10.53 (3.5–24.1)	15.18 (5.8–32.3)	12.67 (6.5–22.3)
Los Angeles	Biobio	3.34 (0.1–18.6)	15.72 (4.3–40.3)	9.03 (2.9–21.1)	3.83 (0.1–21.4)	17.05 (4.6–43.7)	9.91 (3.2–23.2)
Puente Alto	Greater Stgo	7.76 (1.6–22.7)	8.74 (1.8–25.5)	8.22 (3.0–17.9)	9.29 (1.9–27.2)	9.59 (2.0–28.0)	9.43 (3.5–20.6)
Puerto Montt	Los Lagos	5.2 (0.6–18.8)	2.99 (0.1–16.7)	4.17 (0.9–12.2)	5.84 (0.7–21.4)	3.8 (0.1–21.2)	4.9 (1.0–14.5)
Punta Arenas	Magallanes	0.00	4.28 (0.1–23.8)	2.01 (0.1–11.2)	0.00	4.32 (0.1–24.1)	1.99 (0.1–11.1)
Santiago	Greater Stgo	13.73 (5.0–29.9)	5.86 (0.7–21.2)	10.28 (4.4–20.3)	12.26 (4.5–26.8)	5.55 (0.7–20.1)	9.18 (4.0–18.1)
Talca	Maule	5.84 (0.7–21.1)	13.8 (3.8–35.3)	9.49 (3.5–20.7)	5.54 (0.7–20.0)	13.56 (3.7–34.7)	9.22 (3.4–20.1)
Temuco	La Araucanía	3.08 (0.1–17.2)	7.25 (0.9–26.2)	4.99 (1.0–14.6)	3.07 (0.1–17.1)	7.1 (0.9–25.7)	4.92 (1.0–14.4)
Viña del Mar	Valparaiso	7.17 (0.9–25.9)	0.00	3.91 (0.5–14.1)	5.68 (0.7–20.5)	0.00	3.07 (0.4–11.1)

Stgo, Santiago

**Table 5.** Association between 2015 and 2019 annualised incidence of lung cancer and the average concentration of six atmospheric pollutants in Chilean boroughs during the 5 years prior to the estimate. Multiple lineal regression models adjusted by age and HDI.

Lung cancer							
		Unadjusted		Adjusted by age		Adjusted by HDI	
Pollutant		*β* (SE)	*p*-value	*β* (SE)	*p*-value	*β* (SE)	*p*-value
SO_2_							
	General	−1.97 (2.01)	0.431	−1.79 (2.16)	0.560	−2.14 (2.88)	0.593
	Woman	4.65 (4.46)	0.406	5.04 (4.74)	0.480	4.23 (6.37)	0.626
	Male	−10.1 (1.05)	0.011	−10.2 (1.02)	0.063	−10.0 (1.5)	0.092
NO							
	General	0.03 (0.20)	0.899	0.07 (0.06)	0.352	0.05 (0.05)	0.407
	Woman	0.20 (0.26)	0.508	0.233 (0.26)	0.462	0.21 (0.27)	0.508
	Male	−0.19 (0.40)	0.657	−0.14 (0.39)	0.754	0.16 (0.37)	0.705
NO_2_							
	General	0.24 (0.23)	0.362	−0.02 (0.25)	0.929	0.24 (0.35)	0.540
	Woman	0.28 (0.34)	0.453	0.10 (0.47)	0.845	0.05 (0.69)	0.949
	Male	0.19 (0.54)	0.747	−0.19 (0.73)	0.813	−0.63 (0.98)	0.568
O_3_							
	General	0.73 (0.46)	0.175	0.57 (0.50)	0.320	0.23 (0.68)	0.756
	Woman	0.36 (0.56)	0.547	0.17 (0.61)	0.789	−0.15 (0.86)	0.874
	Male	1.17 (1.04)	0.311	1.04 (1.23)	0.445	0.63 (1.67)	0.724
PM_10_							
	General	0.13 (0.11)	0.261	0.11 (0.10)	0.286	0.07 (0.09)	0.473
	Woman	0.14 (0.13)	0.318	0.11 (0.10)	0.309	0.08 (0.12)	0.541
	Male	0.12 (0.20)	0.570	0.11 (0.21)	0.615	0.06 (0.21)	0.790
PM_2.5_							
	General	0.08 (0.13)	0.539	0.07 (0.11)	0.533	0.11 (0.10)	0.270
	Woman	0.02 (0.15)	0.893	0.01 (0.13)	0.971	0.05 (0.13)	0.729
	Male	0.16 (0.24)	0.504	0.15 (0.24)	0.536	0.20 (0.22)	0.390

**Table 6.** Association between 2015 and 2019 annualised incidence of lung adenocarcinoma and the average concentration of six atmospheric pollutants in Chilean boroughs during the 5 years prior to the estimate. Multiple lineal regression model adjusted by age and HDI.

Adenocarcinoma							
		Unadjusted		Adjusted by age		Adjusted by HDI	
Pollutant		*β* (EE)	*p*-value	*β* (EE)	*p*-value	*β* (EE)	*p*-value
SO_2_							
	General	−0.46 (0.89)	0.656	−0.34 (0.16)	0.278	−0.75 (0.76)	0.502
	Woman	3.41 (1.99)	0.228	3.68 (0.42)	0.073	2.78 (1.74)	0.357
	Male	−5.21 (0.50)	0.009	−5.28 (0.23)	0.028	−5.08 (0.54)	0.067
NO							
	General	0.01 (0.17)	0.988	0.04 (0.10)	0.736	0.02 (0.02)	0.382
	Woman	0.11 (0.16)	0.547	0.14 (0.14)	0.422	0.12 (0.12)	0.399
	Male	−0.14 (0.27)	0.645	0.09 (0.23)	0.724	−0.11 (0.19)	0.619
NO_2_							
	General	0.33 (0.15)	0.091	0.15 (0.14)	0.378	−0.04 (0.17)	0.822
	Woman	0.40 (0.15)	0.057	0.30 (0.21)	0.236	0.25 (0.30)	0.468
	Male	0.24 (0.34)	0.517	0.04 (0.44)	0.928	−0.40 (0.56)	0.522
O_3_							
	General	0.81 (0.29)	0.037	0.67 (0.28)	0.075	0.38 (0.37)	0.365
	Woman	0.63 (0.30)	0.086	0.48 (0.28)	0.163	0.22 (0.40)	0.612
	Male	1.03 (0.66)	0.177	0.90 (0.76)	0.302	0.55 (1.03)	0.622
PM_10_							
	General	0.13 (0.08)	0.121	0.11 (0.06)	0.085	0.08 (0.06)	0.187
	Woman	0.14 (0.09)	0.149	0.12 (0.05)	0.055	0.08 (0.06)	0.235
	Male	0.13 (0.13)	0.367	0.12 (0.14)	0.424	0.08 (0.14)	0.561
PM_2.5_							
	General	0.13 (0.10)	0.210	0.12 (0.07)	0.117	0.16 (0.06)	0.023
	Woman	0.07 (0.11)	0.513	0.06 (0.08)	0.460	0.10 (0.08)	0.202
	Male	0.21 (0.16)	0.211	0.19 (0.15)	0.211	0.23 (0.14)	0.123

## References

[1] Cohen AJ (2003). Air pollution and lung cancer: what more do we need to know?. Thorax.

[2] Cohen AJ, Pope CA (1995). Lung cancer and air pollution. Environ Health Perspect.

[3] Pope CA, Burnett RT, Thun MJ (2002). Lung cancer, cardiopulmonary mortality, and long-term exposure to fine particulate air pollution. JAMA.

[4] Wise J (2019). Air pollution report shows health impact. BMJ.

[5] Barrios Casas S, Peña-Cortés F, Osses S (2004). Efectos de la contaminación atmosférica por material particulado en las enfermedades respiratorias agudas en menores de 5 años. Ciencia y Enfermería.

[6] https://gco.iarc.fr/today/online-analysis://gco.iarc.fr/today/online-analysismultibars?v=2018&mode=cancer&mode_population=countries&population=900&populations=900&key=total&sex=0&cancer=39&type=0&statistic=5&prevalence=0&population_group=0&ages_group%5B%5D=0&ages_group%5B%5D=17&nb_items=10&group_cancer=1&include_nmsc=1&include_nmsc_other=1&type_multiple=%257B%2522inc%2522%253Atrue%252C%2522mort%2522%253Atrue%252C%2522prev%2522%253Afalse%257D&orientation=horizontal&type_sort=0&type_nb_items=%257B%2522top%2522%253Atrue%252C%2522bottom%2522%253Afalse%257D&population_group_globocan_id=.

[7] https://gco.iarc.fr/today/data/factsheets/populations/152-chile-fact-sheets.pdf.

[8] Soza-Ried C, Bustamante E, Caglevic C (2019). Oncogenic role of arsenic exposure in lung cancer: a forgotten risk factor. Crit Rev Oncol Hematol.

[9] Plan Nacional del Cáncer 2018–2028. https://www.minsal.cl/wp-content/uploads/2019/01/2019.01.23_PLAN-NACIONAL-DE-CANCER_web.pdf.

[10] Disponible en. https://sinca.mma.gob.cl/.

[11] Bray F, Ferlay J, Soerjomataram I (2018). Global cancer statistics 2018: GLOBOCAN estimates of incidence and mortality worldwide for 36 cancers in 185 countries. CA Cancer J Clin.

[12] Yang P, Allen MA, Aubry MC (2005). Clinical features of 5,628 primary lung cancer patients. Experience at Mayo clinic from 1997 to 2003. Chest.

[13] Gurrola-Díaz CM, González-Santiago AE, Troyo-Sanrománb R (2009). Tipos histológicos y métodos diagnósticos en cáncer pulmonar en un centro hospitalario de tercer nivel. Gac Méd Méx.

[14] Arbex MA, de Paula Santos U, Conceição Martins L (2012). Air pollution and the respiratory system. J Bras Pneumol.

[15] Atkinson RW, Butland BK, Dimitroulopoulou C (2016). Long-term exposure to ambient ozone and mortality: a quantitative systematic review and meta-analysis of evidence from cohort studies. BMJ Open.

[16] White AJ, Bradshaw PT, Herring AH (2016). Exposure to multiple sources of polycyclic aromatic hydrocarbons and breast cancer incidence. Environ Int.

[17] Hamra GB, Hamra GB, Guha N (2014). Outdoor particulate matter exposure and lung cancer: a systematic review and meta-analysis. Environ Health Perspect.

[18] Gharibvand L, Beeson WL, Shavlik D (2017). The association between ambient fine particulate matter and incident adenocarcinoma subtype of lung cáncer. Environ Health.

[19] Park S, Jee SH, Shin HR (2014). Attributable fraction of tobacco smoking on cancer using population-based nationwide cancer incidence and mortality data in Korea. BMC Cancer.

